# Immature ginger reduces triglyceride accumulation by downregulating Acyl CoA carboxylase and phosphoenolpyruvate carboxykinase-1 genes in 3T3-L1 adipocytes

**DOI:** 10.29219/fnr.v67.9126

**Published:** 2023-03-02

**Authors:** Haiwen Li, Ahmed Reza Rafie, Anwar Hamama, Rafat A. Siddiqui

**Affiliations:** 1 Food Chemistry and Nutrition Science Laboratory, Agricultural Research Station, College of Agriculture, Virginia State University, Petersburg, VA, USA; 2 Cooperate Extension, College of Agriculture, Virginia State University, Petersburg, VA, USA; 3 Common Research Laboratory, Agricultural Research Station, College of Agriculture, Virginia State University, Petersburg, VA, USA

**Keywords:** lipid droplets, fatty acids, obesity, glucose uptake, adipogenesis, lipogenesis

## Abstract

**Background:**

Obesity is the underlying risk factor for major metabolism complications, including non-alcoholic-fatty liver disease, atherosclerosis, and cardiovascular disease. The adipose tissue is a vital endocrine organ that plays a role in the synthesis and storage of lipid and, therefore, is a contributory factor to the development and progression of obesity. A growing interest in nutraceuticals suggests that natural products can alleviate the risk factors and may be effective in mitigating obesity.

**Aim:**

The objective of this study was to examine the underlying mechanisms of immature ginger on adipocyte differentiation and lipogenesis in a 3T3-L1 cellular model.

**Methods:**

Ginger samples, extracted in 80% methanol, were dried and resuspended in DMSO at 50 μg/mL as stock solution. For analysis, the extracted samples were further diluted in media. Effects on adipogenesis were evaluated by determining lipid droplet and triglyceride accumulation, whereas effects on lipogenesis were determined by measuring triglyceride contents and fatty acid profile. The expression of key regulatory genes involved in adipogenesis and lipogenesis was also determined.

**Results:**

Our data indicate that the intracellular lipid accumulation decreased significantly by 15 or 25% on treatment with 25 or 50 μg/mL of ginger extract. Consistent with these data, significantly reduced triglyceride levels by 30 or 50% were observed on 25 or 50 μg/mL treatment with ginger extracts, respectively. In addition, ginger treatment significantly inhibited the differentiation-induced *de novo* lipogenesis and Δ9 desaturase activity. Furthermore, ginger treatment reduced adipogenesis genes, *C/ebpβ* and *C/ebpδ*, expression by 47 or 64%, respectively, but significantly increased *Pparγ* expression by 60% and adiponectin by 75%. Ginger extracts had no effect on *Fas* genes but reduced lipogenesis genes, acyl CoA carboxylase (*Acc*) expression by two-fold, and phosphoenolpyruvate carboxy kinase 1 (*Pepck1)* expression by 50%.

**Conclusion:**

Our findings suggest immature ginger can potentially inhibit lipogenesis pathways by limiting the channeling of glucose carbon in fatty acid synthesis by inhibiting the expression of ACC and glycerol production via inhibiting the expression of PEPCK, which consequently inhibits triglyceride formation.

## Popular scientific summary

- This study examined the underlying mechanisms of immature ginger on adipocyte differentiation and lipogenesis in a 3T3-L1 cellular model.
- Our data indicate that the ginger extract reduced intracellular lipid accumulation, triglyceride levels, differentiation-induced *de novo* lipogenesis and Δ9 desaturase activity, and expression of adipogenesis and lipogenesis genes.
- Immature ginger can potentially inhibit lipogenesis pathways by limiting the channeling of glucose carbon in fatty acid synthesis by inhibiting the expression of acetyl-CoA carboxylase (ACC) and glycerol production via inhibiting the expression of phosphoenolpyruvate carboxy kinase (PEPCK), which consequently inhibits triglyceride formation.

Obesity is the most common metabolic disorder worldwide. The epidemic of obesity has nearly tripled since 1975 (1). According to WHO, in 2016, 39% of adults were overweight, and 13% of them were obese globally. In the United States, the occurrence of obesity reached 42.4% in 2017–2018 among adults (2). Obesity is the underlying cause of major metabolism abnormalities. It seriously impacts health and leads to numerous severe complications, including insulin resistance, type 2 diabetes, hypertension, non-alcoholic fatty liver disease, atherosclerosis, and cardiovascular disease (3–7). In addition, obesity is the trigger of various cancers such as colon, breast, endometrium, kidney, and esophagus (adenocarcinoma) cancer (8). These conditions seriously affect the quality of life, cause severe disability, and increase mortality.

Obesity is initiated by the imbalance of energy uptake and its expenditure. The excessive energy is stored in white adipose tissue (WAT) in the form of lipid accumulation (9). The WAT is a complex organ that functions as a reservoir for storing and utilizing the overloaded energy. It plays a key role in energy homeostasis through regulating the secretion of paracrine factors (such as adipokines) and hormones, which trigger metabolic disorders (10). Adipocytes enlarge cell size (hypertrophy) and increase cell number (hyperplasia) in response to the excessive nutrition uptake to compensate for the need for excess lipid storage (11). On the other hand, obesity is closely interconnected with type 2 diabetes. The relationship between obesity and diabetes has been extensively studied. Obesity is a contributing factor that triggers disorders such as insulin resistance and type 2 diabetes (12).

Adipose tissue not only stores passive triglycerides, but it is also a vital endocrine organ. Adipose tissue-infiltrated macrophages are involved in the secretion of a group of proteins called adipokines, including leptin, adiponectin, resistin, visfatin, interleukin-6 (IL-6), monocyte chemotactic protein-1 (MCP-1), and tumor necrosis factor-alpha (TNFα) (13–18). Adipokines lead to a systematic chronic sub-inflammatory state. Obese individuals overproduce non-esterified fatty acids, which consequentially initiate an inflammatory response in adipocytes (17) and secrete the adipokines, which subsequently initiate a low-grade chronic inflammatory state. Adipokines are key regulators of diverse biochemistry processes, especially in fat metabolism, glucose homeostasis, and insulin sensitivity (19). Eventually, the pro-inflammatory cytokines could participate in the development of insulin resistance and increase the onset of metabolic syndrome (20). The inflammatory function of adipokines plays a crucial role in mediating obesity-induced insulinesistance. Adipokines are also key regulators in communicating with other organs and modulating diverse biochemistry processes, such as fatty acid metabolism and glucose homeostasis (19).

The two leading causes of type 2 diabetes are insufficient insulin secretion and insulin resistance that may be mediated by adipose tissue (21, 22). ‘Diabesity’ is a concept linking obesity and diabetes (23). Obese individuals are more likely to develop insulin resistance and are about 10 times more prone to develop type 2 diabetes than normal-weight people (24). The pathogenesis in diabetes involves adipokines-mediated blocking of insulin signaling transduction pathway, causing a progressive development of insulin resistance and eventually leading to type 2 diabetes (18).

Molecular mechanisms of adipogenesis have been well studied using the *in vitro* model of pre-adipocyte cell lines, including 3T3-L1 and 3T3-F442A (25–27). 3T3-L1 cells and 3T3-F442A cell lines were derived from Swiss 3T3 cells, which were initially isolated from mouse embryo in 1962 (28). These cells showed lipid accumulation ability and, on differentiation, displayed mature adipocyte-like characteristics due to the expression of genes for adipogenesis, including C*/ebp* and *Ppar* isoforms (29). However, the 3T3-F442A cells exhibited the greater potential of displaying adipogenic characteristics *in vitro* on growth hormone stimulation (30). Adipocyte differentiation is the procedure where fibroblast-like pre-adipocytes are developed into the well-established adipocytes and accumulate copious lipid droplets (31–33). A highly orchestrated multistep process customizes the transcriptional network and controls the molecular regulation of adipogenesis (34). Several transcription factors are sequentially activated in the adipogenesis transcriptional cascade. Remarkable CCAAT/enhancer binding proteins (C/EBPα, C/EBPβ, and C/EBPδ), peroxisome proliferator-activated receptor-γ (PPARγ), and sterol regulatory element-binding transcription factor 1 (SREBF1) are the upstream genes in promoting adipogenesis (34–40). These factors are essential in promoting the mature adipocyte phenotype. In response to hormonal stimuli, including cortisol, growth hormone, or insulin, C/EBPβ and C/EBPδ are induced immediately, which, in turn, activate PPARγ and C/EBPα (27, 41). The expression of the downstream genes that are necessary for regulating fatty acid storage and glucose metabolism are induced subsequently. The genes involved in the fatty acid synthesis, including the adipocyte protein 2 (ap2), fatty acid synthase (FAS), and acetyl-CoA carboxylase (ACC), are dramatically increased in lipogenesis in adipose tissue (40). These key factors control adipocyte differentiation and fatty acid synthesis, and regulate fatty acid oxidation (33, 42–44).

A lifestyle that includes a high-fat diet and a lack of physical activity is the major factor contributing to obesity. Obesity can be prevented and managed by anti-obesity drugs, such as phentermine and orlistat. They are mainly focused on either suppressing appetite or reducing fat absorption (45). Lifestyle interventions and dietary modification are other approaches to combat obesity (46). However, these strategies are challenging to maintain in the long term. Natural nutraceutical products and new therapeutic interference approaches have been helpful in obesity control. They attenuate obesity by regulating adipogenesis and controlling fat accumulation, the key process in obesity. Polyphenols the major bioactive ingredients from natural food products, including resveratrol (3, 4’, 5-trihydroxystilbene), gingerols, curcumin, and epigallocatechin gallate, have been well studied for their ability to prevent obesity and obesity-related chronic diseases (47–49).

Ginger (*Zingiber officinale Roscoe*) is one of the most popular species used in Asian cuisine for its extraordinary pungency and typical aroma (50). Ginger belongs to an herbaceous perennial plant of the Zingiberaceae family. It is widely used as a nutritional product. Ginger products have been widely used in traditional Chinese and Indian medicine dating back to ancient times (51). Modern remedies also use it as an alternative therapy for treating and maintaining various diseases and health conditions, for instance, cancer, inflammation, non-alcoholic fatty liver disease, metabolic syndrome, especially hypertension, diabetes, and digestive disease (52–55). The major bioactive compounds in ginger are gingerols, shogaols, zingerone, and paradols (56). Their health-promoting effects have been well documented. Nammi demonstrated that ginger extracts significantly decreased body weight, glucose, insulin, and metabolic marker panel, including total cholesterol, LDL cholesterol, triglyceride, free fatty acids, and phospholipids in rats (57). Meanwhile, skeletal muscle fat catabolism and energy expenditure were dramatically increased. Ginger extracts accelerated lipid and glucose metabolism through multiple mechanisms, including regulation of adipogenesis, increasing glucose uptake and energy expenditure, decreasing hepatic lipogenesis, and attenuating the inflammation through the Adenosine monophosphate-kinase (AMPK), PPARs, and Nuclear factor kappa B (NF-kB) signaling pathways (58–61). Active compounds such as 6-gingerol decreased obesity by changing the activities and expression levels of some lipid metabolism markers, such as FAS, ACC, 3-hydroxy-3-methylglutaryl-CoA reductase (HMGCR), lecithin choline acyl transferase (LCAT), and lipoprotein lipase (LPL) (62).

In the previous study, we conducted research to determine the phenolic content and anti-oxidation properties of ginger at different harvesting times. Effects on lipid droplet formation and glucose uptake in HepG2 cells were also examined. Our data indicated that ginger has the highest content of phenolic contents and superior anti-oxidation activity when harvested early (immature ginger). Immature ginger inhibits lipid accumulation and triglyceride content in oleic acid-induced HepG2 cells, significantly inhibits α-amylase enzyme activity, and ameliorates glucose uptake in HepG2 cells (63). Our previous studies suggest that a regular use of ginger can potentially lower incidences of obesity and diabetes. However, how ginger extract inhibits adipogenesis and decreases obesity has not been thoroughly investigated. The objective of this study is to examine the underlying molecular mechanisms on adipogenesis in a 3T3-L1 adipocyte cell model.

## Materials and methods

### Chemicals and regents

Dulbecco’s Modified Eagle’s Medium (DMEM), antibiotic/antimycotic solution, fetal bovine serum (FBS), and 0.25% trypsin with 0.9 mM EDTA were purchased from Invitrogen (Carlsbad, CA). Pre-adipocyte 3T3-L1 cell line was purchased from the American Type Culture Collection (Rockville, MD). Roche Cell Proliferation Reagent WST-1 (4-[3-(4-Iodophenyl)-2-(4-nitro-phenyl)-2H-5-tetrazolio]-1,3-benzene sulfonate), insulin, dexamethasone, isobutylmethyl xanthine (IBMX), and 2-NBDG (2-(N-(7-Nitrobenz-2-oxa-1,3-diazol-4-yl) Amino)-2-Deoxyglucose) were purchased from Sigma-Aldrich (St. Louis, MO). Oil red O staining kit was purchased from Lifeline Cell Technology (Frederick, MD). Triglyceride Colorimetric Assay kit was purchased from Cayman Chemical (Ann Arbor, Michigan). RNeasy Plus Universal Mini Kit, RT2 Easy First Strand Kit, and qPCR SYBR® Green Master-mix were purchased from Qiagen (Hilden, Germany). Polymerase chain reaction primer used in quantitative real-time reverse transcription-polymerase chain reaction (qRT-PCR) was purchased from RealTimePrimers.com or Life Technologies-Thermo Fisher Scientific (Waltham, MA).

### Ginger cultivation and methanol extraction

Chinese white ginger seed-rhizomes harvested in 2016 were planted in one-gallon pots in a greenhouse, and after a 4-month growth, ginger plants were transplanted into high tunnels for continuous growing. Ginger root samples were harvested starting at week 10, cleaned with tap water, and diced into small pieces. After freeze-drying under low pressure (200 mTorr) in a freeze dryer (SP Scientific, Warminster, PA), samples were milled using a Science-ware Bel-Art Microvilli (Pequannock, NJ) and filtered through a 40 μm mesh strainer by shaking (SCO-0330, Pro Shakeron, Scilogex, Dawsonville, GA). The milled ginger powder was extracted with 80% methanol (1:20 g/mL) for 18 h at ambient temperature with 250 × g shaking on Pro Shaker, followed by centrifugation at 2,500 × g at 20°C for 40 min. The methanol extracts were then evaporated in a nitrogen evaporator (Organomation Associates, Inc, Berlin) followed by freeze drying overnight to remove the traces of methanol/water residues. The dried residues were dissolved in DMSO. Ginger extracts were kept under nitrogen and stored at –20°C until further analysis.

### Pre-adipocyte cell culture and cytotoxicity assay

Quantification of cell viability/cytotoxicity was evaluated using a WST-1 assay. The freeze-dried ginger extracts were re-dissolved in DMSO and examined for cytotoxicity on 3T3-L1 pre-adipocyte cells. Cells are initially proliferated in T-75 flasks and maintained in DMEM containing 4 mM L-glutamine, 4,500 mg/L glucose, 1 mM sodium pyruvate, and 1,500 mg/L sodium bicarbonate (ATCC® 30-2002^TM^) with 10% fetal bovine serum and antibiotic/antimycotic solution. Cells were seeded at 1 × 10^4^/well in a 96-well plate and cultured at 37°C in a humidified atmosphere with 5% CO_2_ for 24 h. 3T3-L1 pre-adipocyte cells were then treated with immature ginger extract with concentrations ranging from 6.25 to 200 μg/mL. Cells treated with an equal amount of DMSO were used as the negative control. After incubating cells at 37°C for 24 h, cell viability was determined by adding 10 μL WST-1 and incubating at 37°C for an additional 2 h according to the manufacturer’s protocol. Absorbance at 440 nm, due to reduced WST-1 by mitochondrial dehydrogenases in intact cells, was recorded to estimate % of live cells. All measurements were taken in triplicate with microplate reader Spectra Max M5.

### Intracellular antioxidant effects of ginger extracts in 3T3-L1 cells

A chloromethyl-derivative of 2’, 7’-dichlorodihydrofluorescein diacetate (H_2_DCFDA) and CM-H_2_DCFDA (5’,6’-chloromethyl-2’, 7’-dichlorodihydrofluorescein diacetate) was used as an indicator for the detection of reactive oxygen species (ROS) in 3T3-L1 pre-adipocyte cells. Briefly, cells were proliferated at 1 × 10^4^/well in 96-well plate and maintained at 37°C with 5% CO_2_. Cells were treated with ginger extract at 0, 12.5, 25, 50, 100, 200, 400, and 600 μg/mL concentrations. Cells treated with serum-free culture medium with DMSO were used as the negative control. Followed by incubation at 37°C for 24 h, intracellular antioxidant activity was determined by immediately incubating the cells with the cell-permeant fluorescent probe CM-H2DCFDA at 37˚C for an additional 2 h, as described previously (64).

### Adipocyte differentiation

Adipocyte differentiation of 3T3-L1 pre-adipocyte cells was performed using the cell-based adipogenesis assay kit following the manufacturer’s manual (Abcam, Waltham, MA). In brief, pre-adipocytes were seeded in flat bottom 6-well plates at a density of 1 × 10^6^ cells per well and incubated at 37°C with 5% humidified CO_2_ upon 40–50% confluence. Cells were pre-treated with 0, 25, and 50 μg/mL of immature ginger extracts in a growth medium for 48 h. The pre-treated cells were then transferred to differentiation medium and maintenance media as described previously (65). Adipogenesis of the pre-adipocyte fibroblasts into mature adipocytes was confirmed by Oil Red O staining as described as follows.

### Oil Red O staining

The cellular lipid accumulation during the adipocyte differentiation was determined by Oil Red O staining. After 7 days of treatment with a differentiation medium, an Oil Red staining assay was conducted according to the manufacturer’s manual (Lifeline Cell Technology, Frederic, MD) as previously described (66). After Oil Red-O staining, pink to red stained oil droplets in the differentiated cells were photographed using a phase-contrast inverted microscope (Olympus CKX41, Tokyo, Japan) and a digital camera at 40× magnification. The Oil Red O-stained differentiated cells were dried completely, and Oil Red O staining was extracted with isopropanol 100% and gently mixed for 15–30 min. The supernatant was added to a 96-well plate and quantified at 490 nm with a Spectra Max microplate reader. All experiments were repeated six times.

### Fatty acid analysis

The 3T3-L1 cells, after differentiation, were treated with 0, 25, 50, 75, and 100 μg/mL ginger extracts as described above. After treatment, cells were scraped and collected in a 15 μL centrifuge tube. Cells were washed 1× with PBS by centrifugation at 2,000 × *g*, and the pallets were used for the determination of fatty acid composition using gas chromatography as described previously (67).

### Triglyceride content assay

3T3-L1 pre-adipocyte cells were seeded in six-well plates. Subsequently, cells were pre-treated with 0, 25, and 50 μg/mL of ginger extracts and incubated at 37°C with 5% humidified CO_2_ for 48 h. The cells were then fed with fresh ginger extracts in the differentiation medium as described above and incubated for another 5 days under the same conditions. The medium was changed to DMEM containing 10% fetal bovine serum and 10 μg/mL insulin (Maintenance medium) for differentiation and incubated at 37°C, 95% O_2_, and 5% CO_2_ for 2 days. Whereas the control group was treated with DMEM containing only 10% fetal bovine serum. Differentiated cells with oil droplets were measured for triglyceride content on day 7 after differentiation. The triglyceride content in the cell lysates was quantified using the Cayman Chemical triglyceride colorimetric assay following the manufacturer’s protocol (Ann Arbor, MI) as described (68). Triglyceride content was expressed as mg/dL of lysate (*n* = 5).

### Calculation of fatty acid synthesis enzyme activity

The ratio of 16:0 to 18:2n-6 was used to calculate a *de novo* lipogenesis (DNL) index (69, 70). The ratio of 18:1n-9/18:0 was used to calculate hepatic stearoyl-CoA-1(delta-9) desaturase activity. The ratio of 18:0/16:0 was used to calculate elongase activity (71).

### Glucose uptake assay in 3T3-L1 pre-adipocyte using 2-NBDG

To evaluate the insulin-like or insulin-sensitizing effects of ginger on glucose uptake, the fluorescently labeled deoxy-glucose analog, 2-NBDG, was used for the direct detection of glucose taken up in cells using a Cayman Chemical Glucose Uptake Cell-Based Assay Kit (Ann Arbor, MI) as described previously (72). In brief, 3T3-L1 cells were cultured in 96-well plates at a density of 1 × 10^4^ cells per well, as described above. Cells were then treated with ginger extracts at 0, 25, and 50 μg/mL concentrations for an additional day. The cell culture medium was then changed to glucose-free DMEM medium with or without ginger extracts and further incubated for 8 h. Subsequently, the serum and glucose replete adipocytes were co-cultured with ginger extracts and 2-NBDG at a final concentration of 200 μg/mL for another 16 h. At the end of the treatment, cells were washed twice, and the fluorescence of retained 2-NBDG was measured with a 96-well plate reader (Spectra Max M5, Molecular Devices) at 485 nm (excitation wavelength) and 535 nm (emission wavelength). All experiments were repeated 5 times. The glucose uptake was determined by comparison of 2-NBDG in cells treated with or without ginger extracts.

### RNA isolation and gene expression analysis with qRT-PCR

Total RNAs from all treated samples were prepared using an RNeasy Plus Universal Mini Kit (Qiagen, Hilden, Germany) according to the manufacturer’s instructions. The quantity and quality of total RNA were determined by measuring the absorbance with a Nano Drop spectrophotometer. The RNAs were treated with DNaseI at room temperature for 15 min to remove genomic DNA contamination. First strand complementary DNA (cDNA) was synthesized using 40 ng RNA with RT^2^ First Strand Kit from Qiagen (Qiagen, Hilden, Germany). The gene expression levels were analyzed by Quantitative real-time RT-PCR conducted on the Bio-Rad CFX-96 Real-Time PCR System using RT^2^ SYBR Green Master mix (Bio-Rad Laboratories, Hercules, CA). The Mouse Adipogenesis genes related to the differentiation and maintenance of mature adipocytes were examined for their expression patterns (73, 74). The marker genes for adipogenesis are listed in Table 1. All results were obtained from at least three independent biological repeats. Data were analyzed using the ΔΔ^CT^ method. All gene expressions were normalized using Glyceraldehyde-3-phosphate dehydrogenase (GAPDH) as internal control and expressed as fold changes of threshold cycle (Ct) value relative to control using the 2− ΔΔCt method.

**Table 1:** Sequences of primers used for real-time PC

Gene symbol	Gene name	Primer sequence
*Pparγ*	Peroxisome proliferator-activated receptor γ	5’-CAAGAATACCAAAGTGCGATCAA-3’ 5’-GAGCTGGGTCTTTTCAGAATAATAAG-3’
*C/ebpβ*	CCAAT/enhancer-binding proteins β	5’-AAGCTGAGCGACGAGTACAAGA-3’ 5’-GTCAGCTCCAGCACCTTGTG-3’
*C/ebpδ*	CCAAT/enhancer-binding proteins δ	5’-TTCAGCGCCTACATTGACTC-3’ 5’-GCTTTGTGGTTGCTGTTGAAG-3’
*Acc*	Acetyl-CoA carboxylase	5’-CGATCTATCCGTCGGTGGTCT-3’ 5’-ATCCCTTTCCCTCCTCCTCC-3’
*Pepck1*	Phosphoenolpyruvate carboxykinase 1	5’- AAG GTC ATT TAA GGG CCA TC-3’ 5’-ATC CTC TGA GCT CCA CTC CT-3’
*Adipo*	Adiponectin	5’-TGTTCCTCTTAATCCTGCCCA-3’ 5’-CCAACCTGCACAAGTTCCCTT-3’
*Fas*	Fatty acid synthase	5’-ATCAAGGAGGCCCATTTTGC-3’ 5’-TGTTTCCACTTCTAAACCATGCT-3’
*Gapdh*	Glyceraldehyde-3-phosphate dehydrogenase	5’-ATCCCATCACCATCTTCCAG-3’ 5’-CCATCACGCCACAGTTTCC-3’

### Statistical analysis

Tests were conducted on data reported as mean ± standard deviation (*n* = 3 or 5). Analysis of variance (One Way ANOVA) and Tukey’s HSD post hoc analyses using SPSS Statistics 20 software were used to determine differences among group means. Between groups statistical significance was represented ‘*’ at *P* < 0.05 or ‘**’ at *P* < 0.01. Within groups statistical significance was represented by different letters, indicating that groups are significantly different at *P* < 0.05. Treatments represented with the same letter are not significantly different from each other.

## Results

### Immature ginger extracts inhibited intracellular oxidation in a dose-dependent manner

**Fig. 1:**
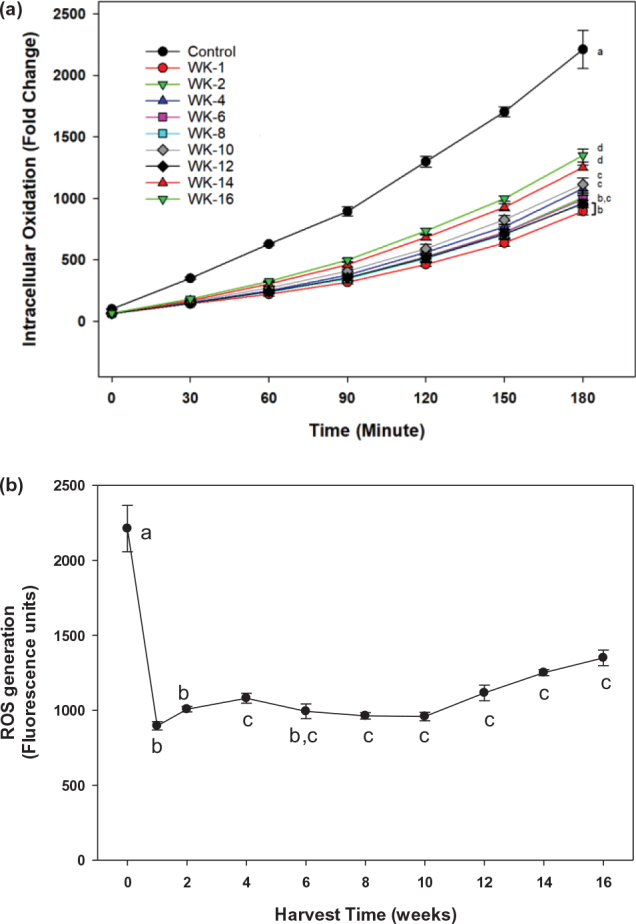
Effects of ginger extract on intracellular oxidation of 3T3-L1 pre-adipocyte cells. (a) Cells were cocultured with 50 μg/mL of ginger extract harvested bi-weekly from week-1 to week-16. The intracellular oxidation was monitored for 3 h using CM-H_2_DCFDA as described in the text. Different symbols in the parenthesis represent (b) replot of data for harvest time at 180 min to determine plateau effect. Values are mean ± SD for at least 3 replications. The statistical analysis was performed using one-way ANOVA (analysis of variance) with Tukey’s HSD post at 180 min of incubation. The statistically significant difference at *P* < 0.05 between groups was represented by different letters compared to control (0 μg/mL).

**Fig. 2:**
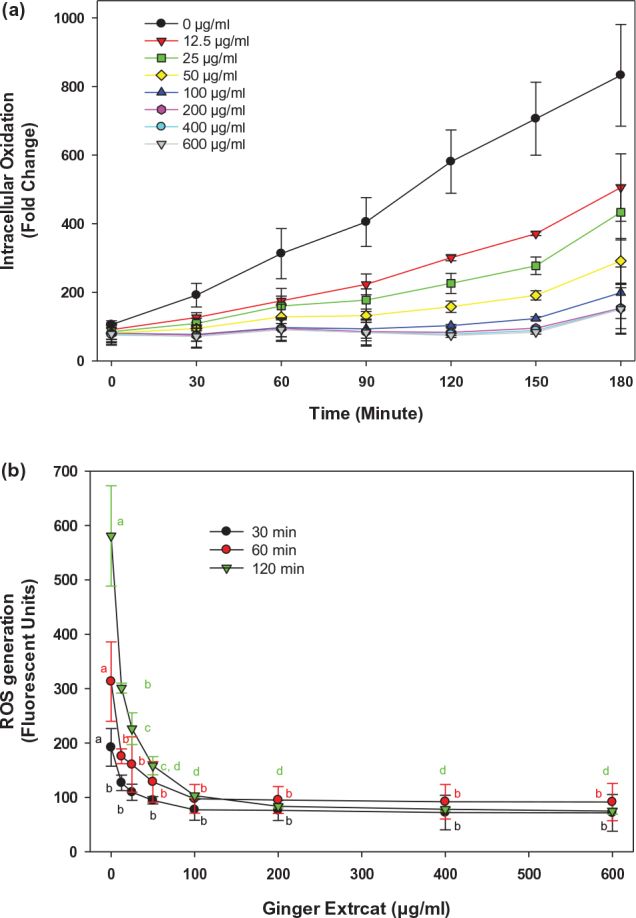
Dose-response effect of ginger extract. (a) Different concentrations of ginger extracts (0, 12.5, 25, 50, 100, 200, 400, and 600 μg/mL) from week 1 harvest were used as described in the legend of Fig. 1. (b) Replot of data at 30, 60, and 120 min to determine IC_50_. Values are mean ± SD for at least 3 replications. Statistical analysis of data in figure b was performed using one-way ANOVA (analysis of variance) with Tukey’s HSD post hoc test. The statistically significant difference at *P* < 0.05 within groups was represented by different letters compared to control (0 μg/mL).

Intracellular ROS generation was evaluated in 3T3-L1 when treated with extracts of ginger harvested at different times during the 16-week period (Fig. 1). The results showed that 50 μg/mL ginger extracts significantly (*P* < 0.05) reduced intracellular ROS levels compared with the control treatment (Fig. 1a). Immature ginger, harvested at the first week, showed highest reduction of intracellular ROS, reducing it by 60% than control treatment. The ROS reduction declined gradually when ginger grew more toward the mature stage, and by week 4, the ROS production was inhibited only by 30%. Harvesting ginger after week 4 has no further significant reduction in ROS generation (Fig. 1b). To examine a dose-dependent on intracellular ROS reduction, the 3T3-L1 cells were exposed to different concentrations of ginger extracts. As shown in Fig. 2a, the intracellular ROS level was reduced by ginger extracts compared to the control treatment, and the ROS reduction showed a dose-dependent effect at concentrations ranging from 12.5 to 100 μg/mL. Replotting this data (Fig. 2b) showed that the significant (*P* < 0.05) reduction of ROS was maximal around 100 μg/mL with IC_50_ ranging from 15 to 20 μg/mL (Fig. 2b). This result is consistent with our previous finding that immature ginger has potentially greater beneficial roles than matured ginger. Recently, there has been an increased interest in cultivating immature ginger across the USA because of its increased culinary demand. Earlier studies have used mature ginger for its health benefits, including anti-obesity effects. However, it is unknown whether immature ginger can similarly induce anti-obesity effects. This study was, therefore, performed using immature ginger for the subsequent experiments.

### Effects of ginger extract on 3T3-L1 cell viability

**Fig. 3:**
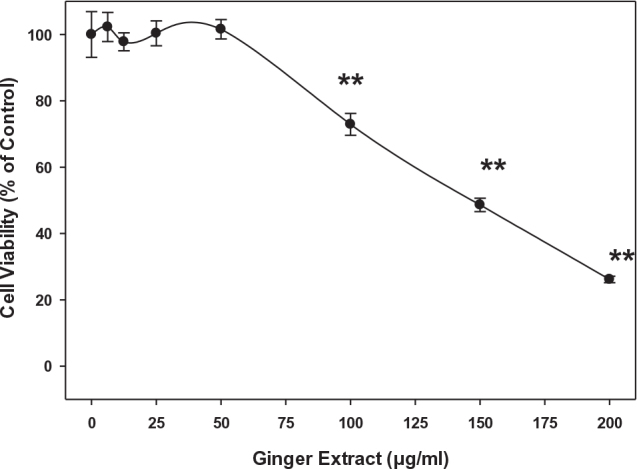
Effects of ginger on 3T3-L1 cells viability. Cells were cultured with 0, 6.25, 12.5, 25, 50, 100, 150, and 200 μg/mL of ginger extract and incubated for 24 h. Cell viability was determined using a WST-1 assay. Values represent mean ± SD for at least three experiments. Statistical analysis was performed using one-way ANOVA (analysis of variance) with Tukey’s HSD post hoc test. **A significant difference at *P* < 0.01, respectively, when compared to control (0 μg/mL).

High concentrations of ginger extracts demonstrated better inhibitive effects on intracellular oxidation in 3T3-L1 cells; however, such measurements were within a relatively short period of time, with only 3-h treatment. Longer treatment by ginger extracts may lead to cytotoxicity to 3T3-L1 cells. To evaluate such potential cytotoxicity of ginger extracts on 3T3-L1 cells, cells were treated with different concentrations of ginger extracts (0, 6.25, 12.5, 25, 50, 100, 150, and 200 μg/mL) for 24 h. As shown in Fig. 3, no statistically significant inhibitory effect was observed on cell viability when 3T3-L1 cells were treated with 50 μg/mL or lower of ginger extracts. In contrast, cell viability decreased by 27% when the cells were treated with 100 μg/mL compared to the control. More than 100 μg/mL of ginger extracts caused a more dramatic decrease in cell viability. Such a significant drop in cell viability (*P* < 0.01) suggests that a high concentration of ginger extracts can eventually damage 3T3-L1 cells. Unless described, all cell-based experiments were, therefore, conducted with ginger extracts at a non-toxic concentration ranging from 0 to 50 μg/mL.

### Immature ginger extracts attenuated lipid accumulation in 3T3-L1 pre-adipocyte

**Fig. 4:**
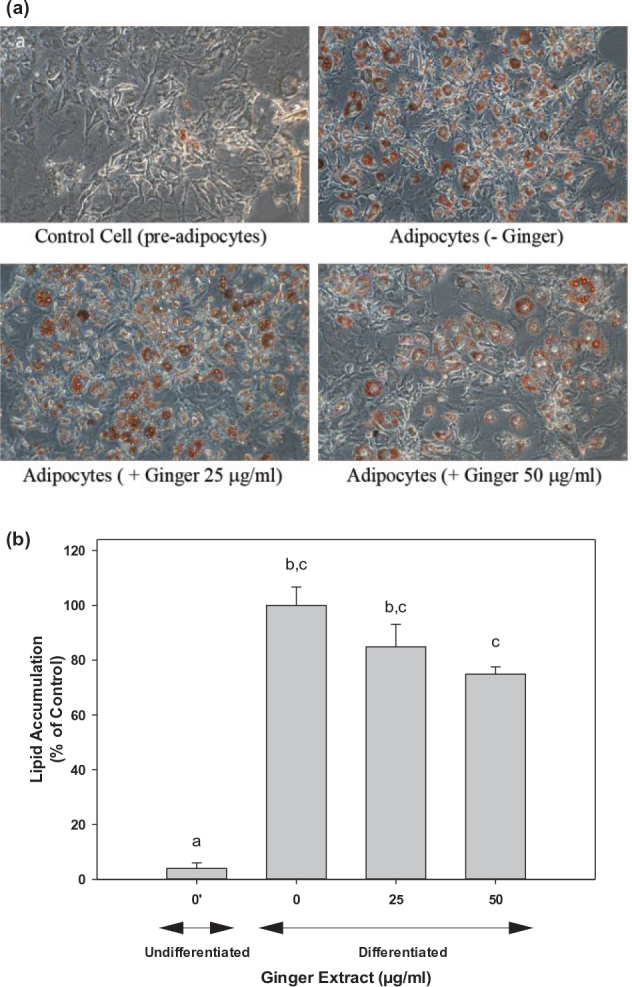
Ginger extract attenuated lipids accumulation in 3T3-L1 pre-adipocyte during adipocyte differentiation. 3T3-L1 pre-adipocyte cells were treated with different ginger extracts treatments (0, 25, and 50 μg/mL). (a) Changes observed in adipocyte differentiation on day 7 of incubation are presented with Oil Red O staining. Cell images were captured at 200× magnification. (b) Cells stained with Oil Red O extracted in propanol, and the optical density was recorded using a microplate reader. Each experiment was performed at least four times, and the individual results are expressed as mean ± SD. One-way ANOVA and Tukey’s HSD post hoc were performed using SPSS Statistics 20 software to determine differences among group means. Within groups, statistically significant difference was represented by different letters, indicating that groups were significantly different at *P* < 0.05. Treatments with the same letter were not significantly different from each other.

The effects of ginger extracts on lipid differentiation were investigated through the induction of adipogenesis in the 3T3-L1 pre-adipocytes. As expected, in the absence of ginger extract treatment, most cells were stained with Oil Red O dye. However, when differentiated cells were treated with ginger extracts, significantly fewer cells were stained by Oil Red O. Furthermore, the cell size for the stained droplets was also significantly smaller than those without ginger treatment (Fig. 4). As compared to the non-differentiated control, the lipid droplet in 3T3-L1 cells gradually increased during the differentiation process. The number and size of the lipid droplets visualized by Oil Red O staining were significantly decreased for both 25 μg/mL and 50 μg/mL immature ginger treatments (Fig. 4a). These results suggested that the ginger extracts attenuate the differentiation and accumulation of lipids. The quantification of the intracellular lipid content is shown in Fig. 4b. The data indicate that the intracellular lipid accumulation decreased by 15% (*P* < 0.05) or 25% (*P* < 0.01) on treatment with 25 or 50 μg/mL of ginger extract, respectively (Fig. 4b). These data strongly suggest that immature ginger can significantly inhibit intracellular lipid accumulation.

### Effect of ginger on lipid metabolism

**Fig. 5:**
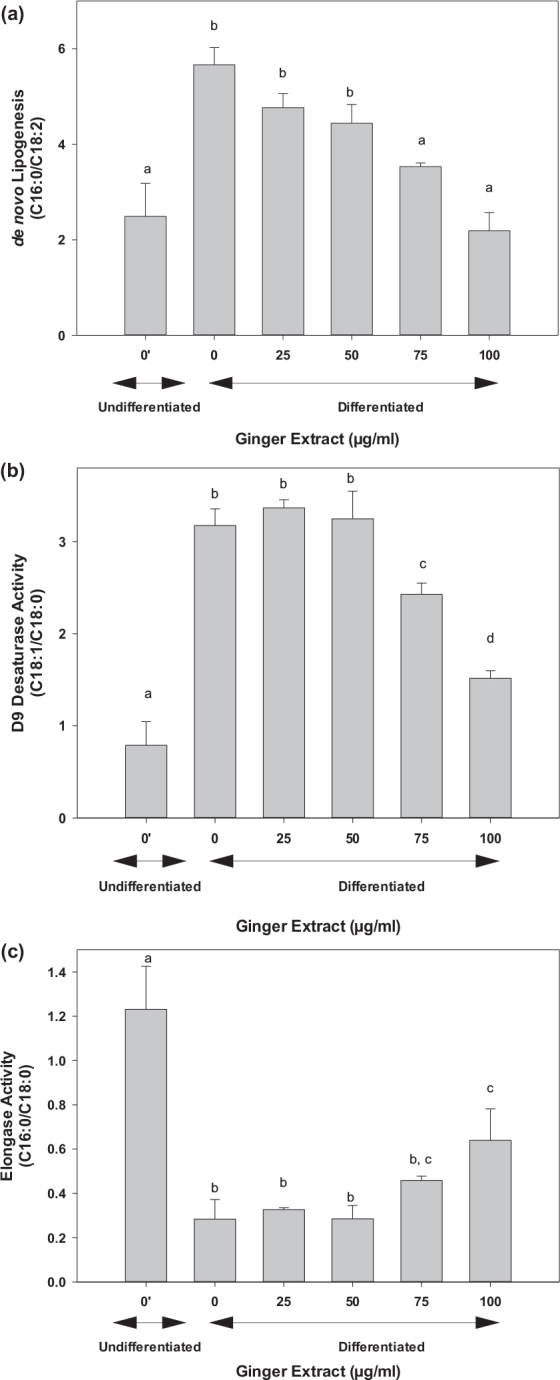
Effect of ginger extract on lipid metabolism. The lipid metabolism was assessed by analyzing the fatty acid profile in cells as described in the text. (a) De novo lipogenesis activity as estimated from C16:0/C18:2 ratios, (b) the Δ9 desaturase activity was calculated using C18:1/C18:0 ratios, and (c) the elongase activity was determined from C18:0/C16:0 ratios. The results are expressed as mean ± SD for at least five replicates. One-way ANOVA and Tukey’s HSD post hoc were performed using SPSS Statistics 20 software to determine differences among group means. Within groups statistical significant differences were represented by different letters, indicating that groups are significantly different at *P* < 0.05. Treatments with the same letter are not significantly different from each other.

**Fig. 6:**
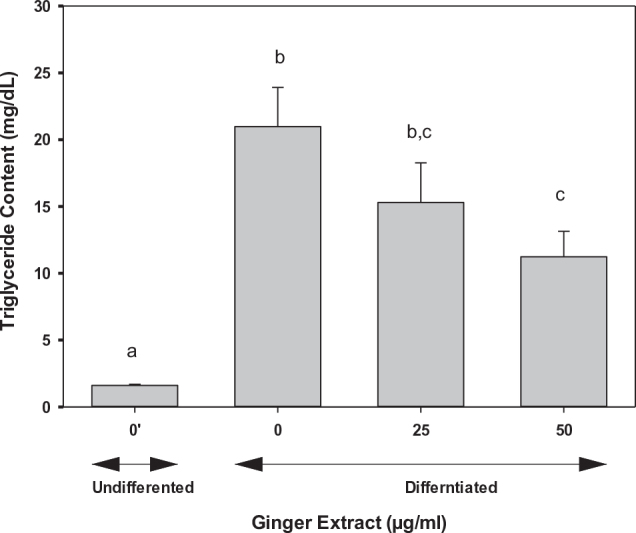
Effects of ginger on triglyceride content in adipocytes. 3T3-L1 cells were treated with 0, 25, and 50 μg/mL of ginger extract. Triglyceride content was quantified using a commercial kit as described in the text. The results represent mean ± SD (*n* = 5). One-way ANOVA and Tukey’s HSD post hoc tests were performed using the SPSS Statistics 20 software to determine differences among group means. Within groups statistical significant differences were represented by different letters, indicating that groups are significantly different at *P* < 0.05. Treatments with the same letter are not significantly different from each other.

Hepatic *de novo* lipogenesis, as assessed using the 16:0/18:2 index, was increased significantly (3.5-fold) in control groups on induction of cell differentiation into adipocytes (Fig. 5a). Treatment with ginger extract reduced the *de novo* lipogenesis to values close to those in the undifferentiated cells at 100 μg/mL (Fig. 5a). Similarly, delta-9 desaturase activity was increased (approximately 3- to 4-fold) on induction of adipogenesis. Treatment of differentiated cells with ginger extract at 25 or 50 μg/mL has no effects, but higher concentrations (75 and 100 μg/mL) significantly reduced the delta-9 desaturase activity (Fig. 5b). Interestingly, induction of adipogenesis caused a significant reduction in elongase activity compared to that of undifferentiated 3T3 pre-adipocytes. Treatment of adipocytes with ginger extract at 25–75 μg/mL has no effect on elongase activity; however, a higher concentration at 100 μg/mL of the ginger extract significantly improved the activity (Fig. 5c). Consistent with these data, we observed a dose-dependent effect of ginger on triglyceride levels in 3T3-L1 cells where a 30% (*P* < 0.05) or 50% (*P* < 0.05) reduction was observed on 25 or 50 μg/mL treatment with ginger extracts (Fig. 6).

### Ginger extracts ameliorated glucose uptake in 3T3-L1 cells

**Fig. 7:**
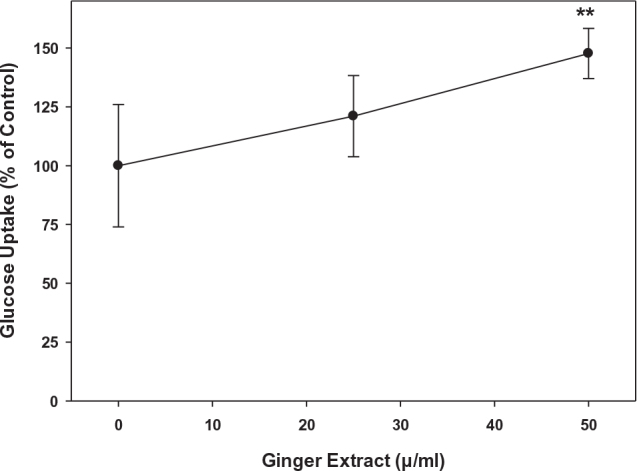
Effects of ginger extracts on glucose uptake in 3T3-L1 pre-adipocyte cells. 3T3-L1 cells were treated with immature Chinese white ginger at 0, 25, and 50 μg/mL. Uptake of 2-NBDG was quantified by measuring fluorescence at 485/650 nm excitation/emission as described in the text. The results represent mean ± SD (*n* = 5). **P < 0.01 compared to 0 μg/mL.

To determine whether ginger extracts affect intracellular glucose uptake, 2-NBDG, a fluorescent glucose analog was introduced into 3T3-L1 adipocytes. Ginger extract stimulated 2-NBDG uptake compared to the control treatment. Glucose uptake in 3T3-L1 cells on 50 μg/mL ginger extract treatment was significantly increased by 48%; however, a significant effect was not detected at 25 μg/mL level even though the trend showed a higher glucose uptake at this concentration (Fig. 7). These results suggest that ginger extracts increased glucose uptake in 3T3-L1 cells, possibly in a dose-dependent manner.

### Ginger extracts influenced the expression of adipogenesis transcription factors and lipogenesis regulatory genes

**Fig. 8:**
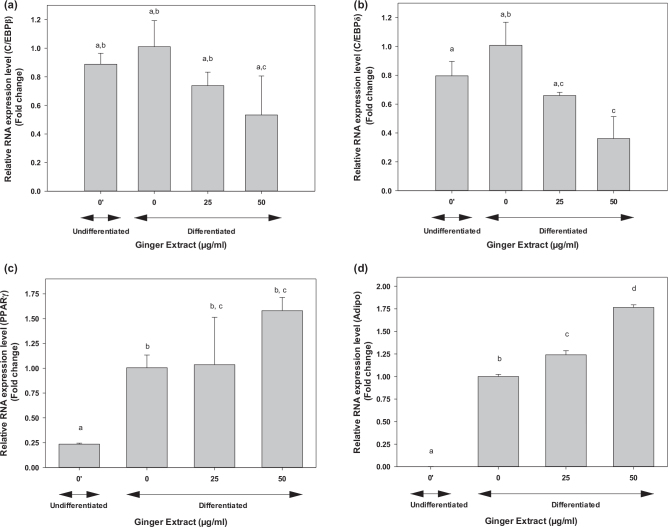
Effect of ginger extract on the expression of genes involved in adipogenesis in 3T3-L1 cells during adipocyte differentiation. The expression of (a) *C/ebpβ*, (b) *C/ebpδ*, (c) *Pparγ*, and (d) adiponectin in 3T3-L1 adipocytes was examined using qRT-PCR using primers reported in Table 1. 3T3-L1 cells were stimulated to differentiation for 5 days as described in the text in the presence of different ginger extract concentrations: 0, 25, and 50 μg/mL. The results represent mean ± SE (*n* = 3). One-way ANOVA and Tukey’s HSD post hoc tests were performed using the SPSS Statistics 20 software to determine differences among group means. Within groups statistical significant differences were represented by different letters, indicating that groups are significantly different at *P* < 0.05. Treatments with the same letter are not significantly different from each other.

Given that ginger extracts significantly reduced oil droplet production in 3T3-L1 cells, real-time qRT-PCR was performed to examine whether ginger extracts affect gene expression related to adipogenesis and lipogenesis at the RNA level. We determined the expression of *C/ebps, ppars*, adiponectin, and *Srebps* transcriptional factors for adipogenesis signaling cascades and *Fas, Acc*, and *Pepck*, regulatory genes for lipogenesis. The mRNA expressions of *C/ebpβ* and *C/ebpδ* were significantly enhanced on adipocyte differentiation compared to undifferentiated cells (Fig. 8). However, ginger extracts significantly inhibited the expression of adipogenesis-related genes compared with the differentiated control adipocytes (*P* < 0.05). A 47 or 64% reduced expression was observed in *C/ebpβ* and *C/ebpδ* genes compared to the control 3T3-L1 cells (Fig. 8a and b). On the other hand, ginger extracts dramatically increased *Pparγ* expression to 1.58-fold (Fig. 8c) and adiponectin by 1.76-fold compared to the control cells (Fig. 8d).

**Fig. 9:**
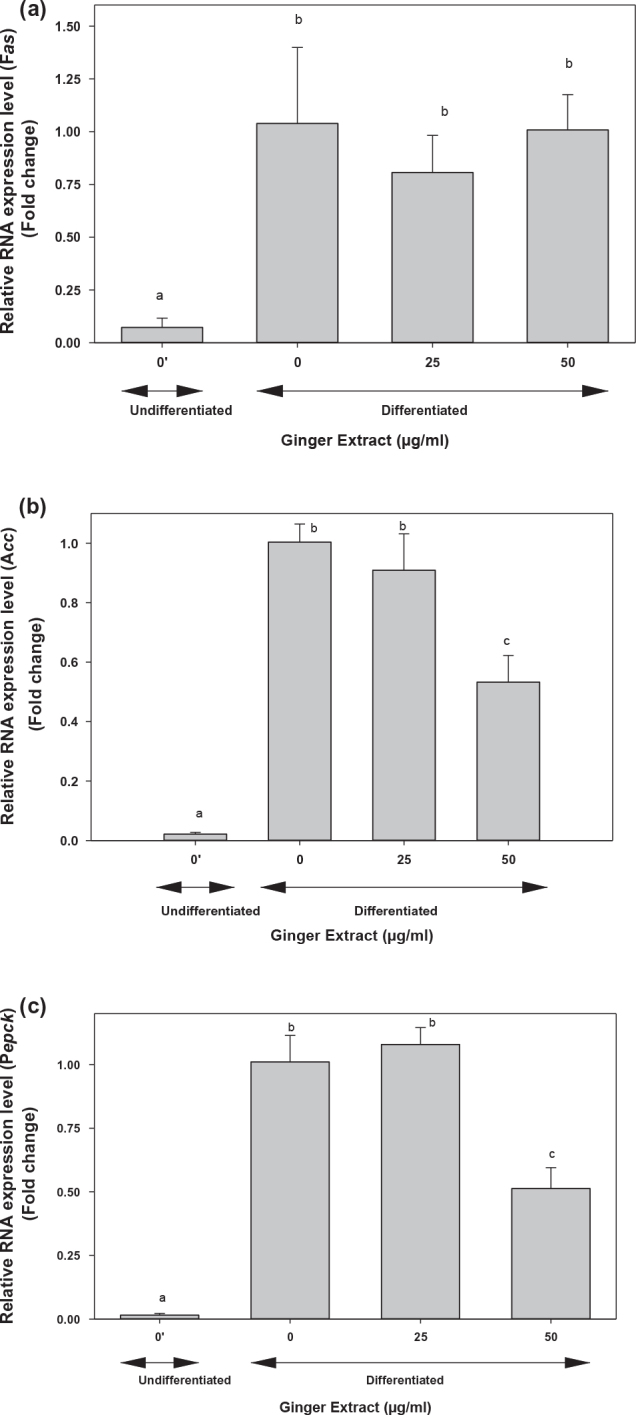
Effect of ginger extract on the expression of genes involved in lipogenesis in 3T3-L1 cells. The expression of (a) *Fas*, (b) *Acc*, and (c) *Pepck1* in 3T3-L1 adipocytes was examined using qRT-PCR using primers reported in Table 1. 3T3-L1 cells were stimulated to differentiation for 5 days as described in the text in the presence of different ginger extract concentrations: 0, 25, and 50 μg/mL. The results represent mean ± SE (*n* = 3). One-way ANOVA and Tukey’s HSD post hoc test were performed using the SPSS Statistics 20 software to determine differences among group means. Within groups statistically significant differences were represented by different letters, indicating that groups are significantly different at *P* < 0.05. Treatments with the same letter are not significantly different from each other.

Ginger extracts (25 or 50 μg/mL) had no effect on *Fas* gene (Fig. 9a) but reduced lipogenesis *Acc* expression by two-fold (Fig. 9b) and *Pepck1* expression by 50% (Fig. 9c) in 3T3-L1 cells compared to the control untreated cells. The expression of *C/ebpα, Srebp1, Lpl*, and *Ap2* was also examined; however, none were affected by 25 or 50 μg mL ginger extracts (data not shown). These results validated that ginger extracts may have anti-obesity potential by control of genes expression involved in adipogenesis and lipogenesis.

## Discussion

Polyphenolic compounds, for example, flavonoids, tannins, and phenolic acids play advantageous roles in inhibiting hyperlipidemia, obesity, type 2 diabetes, and other symptoms of metabolic syndromes (75, 76). Ginger originated in ancient Asia and is widely used as a key food additive worldwide, especially in oriental cuisine. Asian gourmets continue to use it due to its unique pungency and flavor (77). On the other hand, ginger has also been used as herbal medicine to treat diverse ailments for more than 2,000 years (78).

Our previous study evaluated polyphenolic contents and *in vitro* anti-oxidation properties of ginger harvested at different stages. We demonstrated that immature ginger has the highest content of total polyphenols and its major constituents, including 6-gingerol, 6-paradol, and 6-shogaol (63). Its antioxidant activity was also maintained highest for immature ginger and progressively reduced down to 50% as ginger matures. To unveil the antioxidant capacity at the different maturity stages, the 3T3-L1 pre-adipocyte system was used to investigate the intracellular antioxidant capacity of gingers during the 16-week harvest period. Similar to our *in vitro* observations, immature ginger also exhibited the highest antioxidant activity in 3T3-L1 cells in a dose-dependent pattern. The intracellular antioxidant capacity gradually decreased with the continuous ginger maturity (Fig. 1). Antioxidant properties of ginger were also reported in the animal model. In STZ-induced diabetic rats, the ginger extract significantly increased the activities of antioxidant biomarkers: superoxide dismutase (SOD), catalase (CAT), glutathione peroxidase (GPx), and glutathione reductase (GR) and glutathione (GSH) (79). Ginger contains various notable bioactive ingredients, such as polyphenols and flavonoids, and exhibits numerous health-promoting capabilities. Gingerols, paradols, zingerones, and shogaols are major bioactive pungent compositions in ginger (80). Studies have shown that bioactive compounds in ginger exhibited antioxidant activity via the nuclear factor erythroid 2-related factor 2 (Nrf2) signaling pathway, modulated levels of intracellular glutathione/glutathione disulfide, and increased expression of heme oxygenase-1 (HO-1), metallothionein 1, aldo-keto reductase family 1 member B10, ferritin light chain, and γ-glutamyltransferase-like activity 4 (81). Pro-adipogenic effects of ROS have been found in human mesenchymal stem cells (82, 83). Studies have also shown that during adipocyte differentiation of 3T3-L1, a marked increase in ROS production occurred, and scavenging the ROS production inhibited the adipogenesis process (83, 84). It is, therefore, possible that ginger, through its effects on scavenging ROS, may have played in inhibiting adipogenesis processes via its effects on Nrf2 signaling pathway.

As evident from the inhibition of lipid droplet accumulation (Fig. 4) and triglyceride content (Fig. 6), the ginger extract inhibited both adipogenesis and lipogenesis. We further analyzed fatty acid composition to assess lipid metabolism. Our data clearly indicated that ginger reversed processes that are involved in lipogenesis, including *de novo* lipogenesis activity, delta-9 desaturase, and elongation activity (Fig. 5). Consistent with our observation, numerous *in vitro* and *in vivo* studies reported that dietary ginger battled obesity through improving lipid metabolism. Ginger extracts increased glucose uptake and enhanced glucose transportation in L6 skeletal muscle cell line (85). Supplementation of ginger increased fat metabolism, decreased blood lipids profile, reduced the low-density lipoprotein cholesterol, and increased high-density lipoprotein cholesterol in a rat model (86). Ginger extracts also suppressed adipogenesis in pre-adipocytes and inhibited lipid accumulation in mature adipocytes (55). All these observations strongly suggest that ginger can effectively prevent obesity. Our data on fatty acid metabolism showed a dose-dependent effect; however, the significant effects were observed at higher doses, where we typically observed a slight cytotoxic effect of 15–25% at 75–100 μg/mL of ginger extract. The cytotoxic effect of ginger on 3T3-L1 may be due to its direct exposure under *in vitro* conditions. The Food and Drug Administration (FDA) of the United States has recognized ginger as a safe food supplement (87), and several studies have reported no general toxicity in animals and humans (88–95), even at higher doses of 2 g/kg body weight in rats on continuous daily treatment for 5 weeks (92). However, taking large doses on an empty stomach can cause heartburn and abdominal discomfort (96, 97). Excess ginger intake may also cause anti-platelet effects in some people (98).

Although ours and several other studies suggest ginger effect on adipogenesis and lipogenesis, however, the plausible mechanism of ginger on lipid metabolism has not been fully explored. The 3T3-L1 pre-adipocytes are one of the cellular models used to explore the mechanism of adipogenesis (pre-adipocytes differentiation) and lipogenesis (triglycerides accumulation). When these cells were induced with differentiation reagent (3-isobutyl-1-methylxanthine, dexamethasone, and insulin), they subsequently differentiated to matured adipocytes. Transcriptional factors, C/EBPβ and C/EBPδ, are important adipogenic factors, and these are induced promptly by adding the adipogenic stimuli (99). Several studies have demonstrated that inhibiting C/EBPβ and C/EBPδ expression during adipogenesis increases the potential anti-obesity capacity. This is mainly due to C/EBPβ and C/EBPδ downregulation of the major adipocyte-specific genes in fatty acid metabolism, such as FAS, ACC, LPL, and PEPCK (62, 100). A study demonstrated that 6-gingerol in ginger remarkably reduced expression of the lipid metabolism biomarker in HFD-induced obese rats. The expressions of FAS, ACC, and LPL genes were significantly reduced, and body weight gain, serum glucose, insulin levels, and insulin resistance were also significantly inhibited by 6-gingerol (62).

The present study examined the molecular mechanisms underlying immature ginger’s hypo-lipidemic effects on adipogenesis and lipogenesis as potential anti-obesity effects. As mentioned above, ginger also affects the metabolism of glucose and fats and, therefore, can also modulate energy metabolism for its anti-obesity effects. During the present investigation, we focused on studying lipid formation and its storage in adipose tissues. We, therefore, did not study the effect of ginger on fat utilization and energy metabolism as these metabolic processes are mediated by the interaction of multi-organs/tissues and were beyond the scope of this study.

In our study, lipid droplets were visualized when the pre-adipocyte cells were induced for differentiation. Both the size and density of the oil droplets were significantly decreased when cells were cocultured with ginger extracts. At the transcriptional level, immature ginger extracts attenuated the expression of both *C/ebpβ* and *C/ebpδ* in the differentiated 3T3-L1 cells (Fig. 8). In addition, *acc* and *pepck1* genes, involved in fatty acid metabolisms, were downregulated by immature ginger extracts (Fig. 9). As mentioned above, the downregulation of these mediators at gene and protein expression has been well characterized (59, 89); we, therefore, used biochemical characterization of lipogenesis by measuring FA profile to validate the molecular mechanism. Taken together, our findings strongly suggest that immature ginger has potential anti-obesity effects in 3T3-L1 cells through suppressing adipogenesis via down-regulating expression of *C/ebpβ* and *C/ebpδ*, and lipogenesis via down-regulating expression of *Acc* and *Pepck* genes. We also measured glucose uptake by pre-adipocytes to determine if the decrease in lipogenesis was due to inhibition of glucose uptake. In contrast, we found that ginger extract stimulated glucose uptake in pre-adipocytes. Consistent with our observation, gingerols, the most abundant bioactive compound in ginger, have also previously been shown to increase glucose uptake in 3T3-L1 adipocytes (101). Studies have shown that the stimulated glucose uptake was mediated through the activation of AMPK phosphorylation in 3T3-L1 adipocytes (102). Activation of AMP has shown to downregulate genes involved in lipogenesis (including expression of liver X receptor-α, SREBP-1c, and its target genes, including acetyl-CoA carboxylase, FAS, stearoyl-CoA desaturase 1, and acyl-CoA:diacylglycerol acyltransferase), whereas it has upregulated gene expression of fatty acid β-oxidation (PPARα, CPT1, Acox1, and Prkaa1) (59, 103). Furthermore, studies have shown that ginger significantly increased the activities of glucokinase, phosphofructokinase, and pyruvate kinase in diabetic rats (104). These data suggest that ginger can channel enhanced glucose uptake away from lipogenesis into glucose oxidation and, therefore, may play an important role in regulating diabesity.

It is shown that C/EBPα and PPARγ, the two master adipogenic transcription factors, are successively induced during the differentiation process (37, 38, 105, 106). Many reports suggested that ginger extract treatments significantly downregulated both C/EBPα and PPARγ expressions (60, 107). Interestingly, our results indicated that the ginger extract did not affect *C/ebpα* expression and, on the contrary, significantly upregulated *Pparγ* expression (Fig. 7c). These results may suggest that ginger suppressed *C/ebpβ* and *C/ebpδ* expressions through a pathway independent of *Pparγ* and *C/ebpα* expressions. Similar studies with a hydrophobic fraction of ginger extracts (especially 6-gingerol) showed that ginger extracts suppressed lipid accumulation by inhibiting adipogenic gene expression, but the expression of PPARγ at the protein level was increased (48). PPARγ acts as a central character in the network of fat metabolism, glucose homeostasis, inflammation, and other biochemical processes (108). Its expression needs to be balanced during the differentiation process. For example, when 3T3-L1 cells differentiate, increased induction of brown adipocytes was observed. It is found that 6-gingerol can promote the browning of white adipocytes, likely through the AMPK pathway where PPARγ was greatly increased. Lipid metabolism modulated by ginger is a precise network correlated with a different signaling pathway, including PPARs, AMPK, and NF-κB signaling pathways (55). Since we observed inhibition of lipogenesis at relatively higher doses of ginger extract, it is possible that a higher dose of ginger may have inhibited PPARγ and, consequently, lipogenesis. Because of the cytotoxic effects of higher ginger doses, unlike stable fatty acids, it was difficult to analyze less stable mRNA or protein expression for PPARγ. Nevertheless, how ginger is involved in regulating the complex cross-network during differentiation and lipogenesis processes warrants further study.

The WAT-derived adipokine, adiponectin, is a novel therapeutic target for metabolic syndrome. Adiponectin plays a crucial role in glucose homeostasis (109). Adiponectin reduces plasma glucose levels in mice subjected to high-fat diets (110) and directly regulates glucose metabolism in C57BL6J mice (111). Adiponectin also functions as an insulin sensitizer, and its overexpression can improve insulin sensitivity (112). Adiponectin controls the complex signal transduction involved in the insulin signaling pathway. The circulating levels of adiponectin were decreased in obesity-induced insulin resistance mice (113). Adiponectin triggers a cascade of multiple signaling events, including protein synthesis, lipogenesis, glucose metabolism, glycogen synthesis, and reduced lipolysis and gluconeogenesis (7). Impairment of adiponectin causes numerous obesity-related diseases (114). The prolonged overexpression of adiponectin leads to a massive increase in subcutaneous fat and protects against diet-induced insulin resistance (115).

The functional association between PPARγ and adiponectin has also been demonstrated. These two proteins have overlapping functions on downstream metabolic pathways (116, 117). Adiponectin is regulated by PPARγ in controlling its secretion and metabolism. Changes in PPARγ activity alter adiponectin hormonal levels. The comprehensive crosslink between PPARγ and adiponectin was a focus of many studies. Thiazolidinediones (TZD) compounds (troglitazone, rosiglitazone, and pioglitazone) are the commonly used first line anti-diabetes medicines. Their mode of action in correcting diabetic-related metabolic disorders is through the regulation of PPARγ (118–120). TZD is a high-affinity ligand that selectively targets PPARγ and subsequently induces adiponectin production. When patients were treated with TZD, the circulating levels of adiponectin were increased up to three-fold (121). By comparing a TZD (pioglitazone) to a non-TZD (e.g. non-PPARγ) treatment (the sulfonylurea glimepiride), the beneficial lipoprotein changes associated with TZD treatment were due at least in part to increased expression of adiponectin in the TZD treated subjects (122). Adiponectin consequently acts on multiple target tissues, ameliorates fatty acid oxidation, reduces intracellular lipid levels, and improves insulin sensitivity. In the present study, both PPARγ and adiponectin were significantly upregulated by immature ginger treatments. Consistent with their overexpression, the glucose uptake was increased by 47.7% in 3T3-L1 cells by the ginger extract treatment (Fig. 6).

Although it is important to test individual bioactive components present in ginger, during this study we used whole ginger extract instead of the individual chemical components, as our focus was to investigate the effect of ginger as a food with different components that may act synergistically in their anti-obesity effects. However, we plan to test individual components of the processes of adipogenesis and lipogenesis in the near future.

## Conclusions

**Fig. 10:**
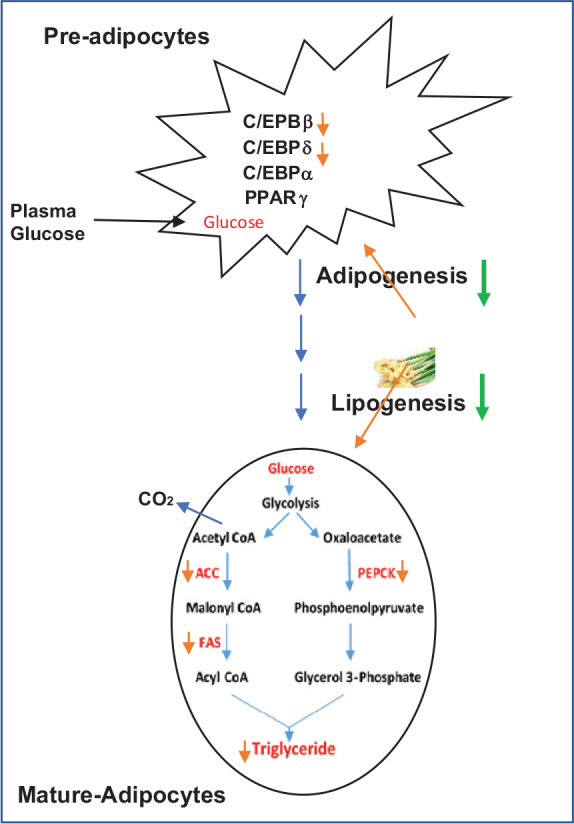
Potential targets of ginger on adipogenesis and lipogenesis to regulate obesity. Ginger can potentially inhibit adipogenesis by reducing the expression of C/EBPb and C/EBPd genes, consequently preventing adipocyte maturation. Ginger can also potentially inhibit the utilization of glucose into lipogenesis pathways by limiting the channeling of glucose carbon in fatty acid synthesis via inhibiting the expression of Acyl CoA Carboxylase (ACC) and Fatty Acid Synthase (FAS) and into glycerol production, and via inhibiting the expression of Phospho-Enol-Pyruvate Carboxy Kinase (PEPCK), which consequently inhibits triglyceride formation. The stimulated glucose uptake by ginger may be converted to CO_2_.

Collectively, our findings shed a light on the potential anti-obesity effects of ginger extracts. The immature ginger extracts modulated the energy metabolism and lipid accumulation in a cellular model. The data suggest that immature ginger may act by regulating cellular differentiation of pre-adipocytes, which makes it suitable as a potential therapeutic agent for obesity. Our results revealed the potential capability of immature ginger in reducing adipogenesis through decreasing the expression of C/EBPβ and lipogenesis through fatty acid synthesis genes, including ACC and PEPCK (Fig. 10). Our study warrants further investigations of the molecular mechanism of the potential capacity of ginger on the anti-obesity effect in an *in vivo* mouse model. The effective concentration of 25–50 μg/mL is equivalent to 400–800 μg of fresh ginger. Considering 4.5L of blood, about 2–4 g of ginger consumption can achieve this level if it is fully absorbed. In reality, the effective amount of intake would be much higher depending on its absorption, bioavailability, and excretion. A higher intake can be achieved by consuming dried powder or extracts. Further research on ginger absorption, bioavailability, and excretion is needed to estimate the daily consumption of ginger to achieve the desired concentration. As mentioned above, ginger is a safe food supplement. Fresh ginger can be used as a tea with honey or can be chopped and added to foods, soups, or salads. It is estimated that a one-fourth inch slice of fresh ginger root is approximately 10 g. This is equivalent to 1 to 2 g of a dry powder form of ginger, which is a more concentrated form found in commercial capsules.

## Data Availability

The data presented in this study are available on request from the corresponding author.
